# Intranasal Administration of Lentiviral miR-135a Regulates Mast Cell and Allergen-Induced Inflammation by Targeting GATA-3

**DOI:** 10.1371/journal.pone.0139322

**Published:** 2015-09-29

**Authors:** Yu-Qin Deng, Ya-Qi Yang, Shui-Bin Wang, Fen Li, Meng-Zhi Liu, Qing-Quan Hua, Ze-Zhang Tao

**Affiliations:** 1 Department of Otolaryngology-Head and Neck Surgery, Renmin Hospital of Wuhan University, Wuhan, Hubei, China; 2 Department of Otolaryngology-Head and Neck Surgery, Yichang Yiling Hospital, Yichang, Hubei, China; Mie University Graduate School of Medicine, JAPAN

## Abstract

Mast cell (MC) degranulation is the foundation of the acute phase of allergic rhinitis (AR). Previously, downregulation of GATA binding protein 3 (GATA-3) was shown to suppress MC activation in an AR mouse model. Binding of microRNA-135a (miR-135a) to GATA-3 was also observed, and overexpression of this miRNA decreased GATA-3 mRNA and protein expression. However, the effects of miR-135a on MCs during AR are currently unknown. In the present study, we utilized a lentiviral (LV) vector to intranasally administer miR-135a to ovalbumin (OVA)-sensitized AR mice. Following miR-135a treatment, the total serum IgE concentration observed during AR was significantly reduced. In the nasal mucosa, the expression of T-box expressed in T cells (T-bet) was higher, whereas that of GATA-3 was lower in the AR mice following miRNA treatment. Notably, during AR, the ratio of type 1 T-helper cells (Th1) to type 2 (Th2) cells in the spleen is unbalanced, favoring Th2. However, administering miR-135a to the AR mice appeared to balance this ratio by increasing and decreasing the percentage of Th1 and Th2 cells, respectively. MiR-135a also appeared to strongly suppress the infiltration of eosinophils and MCs into the nasal mucosa, and it was specifically localized in the MCs, suggesting that its influence is modulated through regulation of GATA-3 in these cells. Additional work identifying the full therapeutic potential of miR-135a in the treatment of AR and diseases involving allergen-induced inflammation is warranted.

## Introduction

Allergen-induced inflammation of the upper airway, also known as allergic rhinitis (AR), is characterized by a variety of symptoms, all of which are caused by an inappropriate or exaggerated immune reaction involving numerous cytokines and pro-inflammatory factors. Mast cells (MCs), specifically the granules in their cytoplasm that are known to contain a variety of powerful biologically active inflammatory mediators, have been shown to play a particularly critical role in AR. In fact, MC degranulation is the foundation of the acute phase of AR, whereby the percentage of MC degranulation reflects the degree of the allergic reaction for the patient [1,2]. Notably, although the transcription factor GATA-3 is highly expressed in Th2 cells and is essential for the differentiation of these cells, GATA-3 expression and function are not limited to Th2 cells. GATA-3 transcripts are relatively abundantly expressed in eosinophils and mast cells [3]. GATA family members, particularly GATA-3, may be critical for “hard-wiring” of a common program of gene expression in mast cells and T helper type 2 cells [4].

Understanding the regulatory pathways involved in gene expression during AR-related inflammation, including that of GATA-3, is essential to provide potential therapeutic targets. In the last two decades, microRNAs (miRNAs or miRs) have been shown to suppress gene expression at the post-transcriptional level by base pairing to the 3′-untranslated region (3′-UTR) of their target mRNA, thus inhibiting protein translation and/or inducing degradation [5–6]. Furthermore, miRNAs are increasingly being regarded as important regulators during development and normal immune system activities as well as during abnormal cellular events, including extreme inflammation [7–9]. Notably, a previous computational investigation utilizing bioinformatics data indicated that simplex miRNA, including those in the miR-135 family, may have the ability to regulate the immune response by targeting GATA-3 and regulating the biased differentiation of T-helper 2 (Th2) cells observed during AR [10]. Moreover, we previously analyzed the 3′-UTR of murine GATA-3, focusing on the target sequences of the miR-135 family, using TargetScan and found a binding site specific for miR-135a, a microRNA that appears to be downregulated during AR [11]. In this prior work, we also overexpressed miR-135a in a mouse model of AR by administering a miR-135a mimic to induce native expression. This overexpression resulted in the downregulation of GATA-3 mRNA and protein expression, while upregulating native miR-135a and T-box expressed in T cells (T-bet) mRNA and protein in the nasal mucosa. Taken together, these data indicate that targeting of GATA-3 with miR-135a is an effective option for correcting the Th1/Th2 imbalance associated with acute AR. Furthermore, we suspect that these effects are ultimately caused by miR-135a-mediated regulation of MC via GATA-3; however, this mechanism has not been fully investigated.

In the present study, we sought to identify the effects of miR-135a-mediated GATA-3 regulation on the MCs of mice with AR to further understand the pathogenesis of this common allergen-induced immune response. To this end, we utilized lentiviral (LV) miR-135a to infect ovalbumin (OVA)-sensitized AR mice, which were then used to determine the localization and effects of miR-135a overexpression on GATA-3 and MC in AR mice.

## Materials and Methods

### Animals

Pathogen-free male BALB/c mice (6–8 weeks old, weighing 18–20 g) were purchased from the Wuhan Institute of Biological Products Co., Ltd (permission number: SCXK-2008-0003, Wuhan, China), and divided into four experimental groups (n = 12 each): normal (control), AR (AR-induced, treated with saline), positive (AR-induced, treated with LV miR-135a), and negative (AR-induced, treated with an empty LV vector). All animals were housed in the campus animal facility under standard conditions, including typical light/dark cycles, average temperature (18–22°C), and mild humidity (50–60%), and were given food and water *ad libitum*. All procedures were approved by the Animal Ethics Committee at Renmin Hospital, Wuhan University (Approval: S0271402201A).

### AR induction

Following a five-day adaptation period, AR was induced in the AR, negative, and positive groups as previously described [12]. Briefly, BALB/c mice were sensitized with ovalbumin (OVA) through a combination of intraperitoneal injection and intranasal challenge in an awake condition.

**Fig 1:**
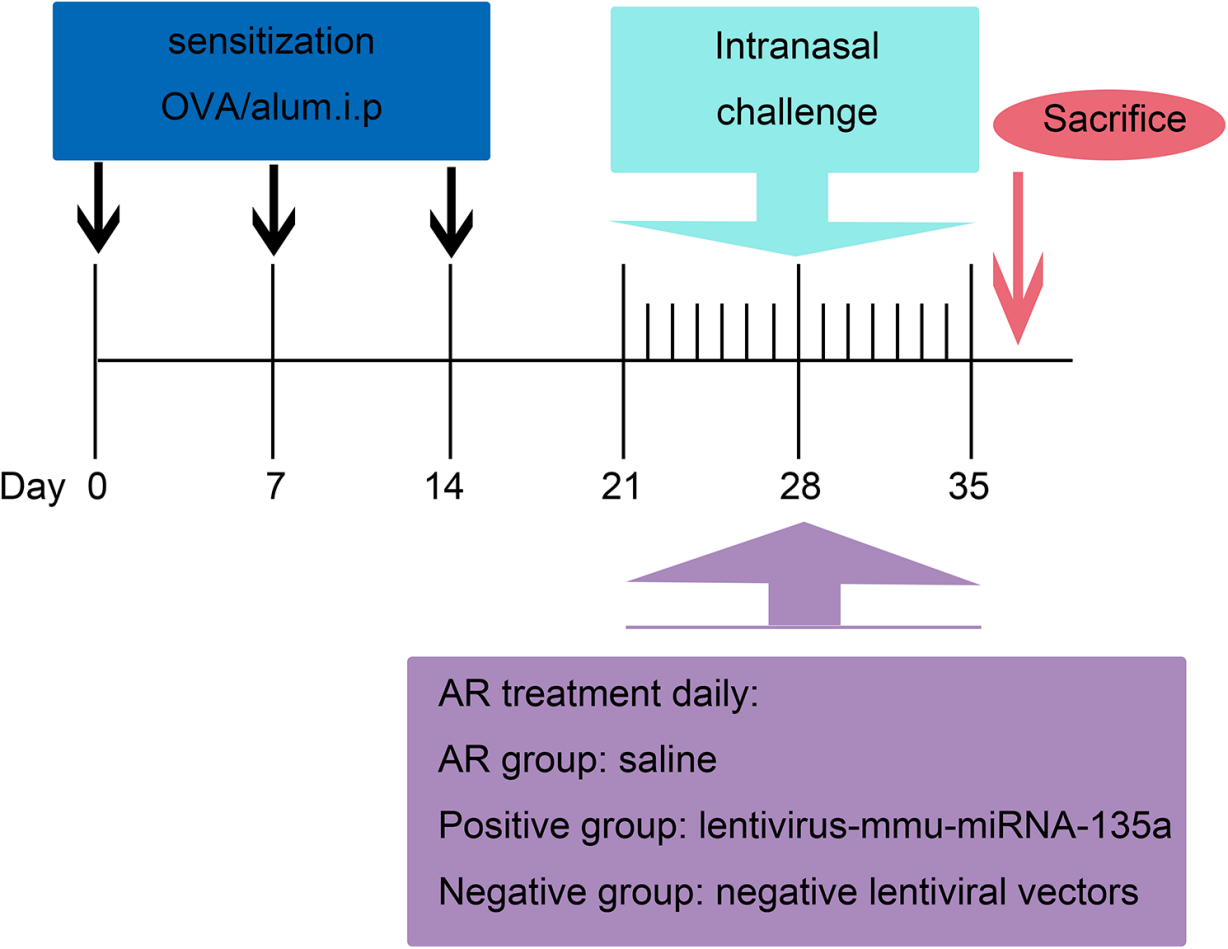
Sensitization and treatment protocol. Mice in the AR, positive, and negative groups were sensitized with 100 μg of ovalbumin (OVA) and 1 mg of aluminum hydroxide on days 0, 7, and 14. On days 21–35, these mice were intranasally administered 40 μl of saline containing 400 μg of OVA (20 μl per nostril) for secondary immunization. Furthermore, within 3 h of each treatment on days 21–35, the AR group was treated with saline, whereas the positive and negative groups were nasally administered 2 × 10^6^ infectious units (IFUs) of LV miR-135a and 2 × 10^6^ IFUs of an empty LV vector, respectively.

The mice were intraperitoneally administered 300 μl of saline containing 100 μg OVA and 1 mg of aluminum hydroxide (Sigma-Aldrich, St. Louis, MO, USA) on days 0, 7, and 14 to promote primary sensitization. On days 21–35, mice in these three groups were intranasally sensitized with 40 μl of saline containing 400 μg of OVA (20 μl per nostril) for secondary immunization. The normal group was intraperitoneally injected with 300 μl of saline on days 0, 7, and 14, and then treated intranasally with 40 μl of saline on days 21–35 after the initial treatment (Fig 1).

### LV vector construction and administration

The LV vector containing mmu-miR-135a was constructed at the GeneChem Company (Shanghai, China). All of the LV vectors utilized in this study expressed enhanced green fluorescent protein (GFP), which enabled fluorescent localization. Following construction, 2 × 10^6^ IFUs of the LV miR-135a were intranasally administered to mice that were fully awake in the positive group within 3 h of AR induction on days 21–35 according to a previously established protocol [13]. The negative group was treated with 2 × 10^6^ IFUs of an empty LV vector, whereas the normal group was treated with saline (Fig 1).

### Measurement of IgE

Blood was collected from the orbital venous plexus of anesthetized mice 24 h after the final intranasal sensitization. The samples were centrifuged for 10 min at 1000 × rpm to isolate the serum and stored at −80°C until use. Total serum IgE was determined using a Mouse IgE ELISA Kit (Cusabio Biotech Co., Ltd, Wuhan, China).

### Quantitative mRNA analysis

Total RNA from the nasal mucosa was extracted using TRIzol reagent (Invitrogen, Carlsbad, CA, USA). The RNA (2 μg) was then reverse transcribed using a RevertAid First Strand cDNA Synthesis Kit (Thermo Scientific^TM^, NY, USA) according to the manufacturer’s instruction. The expression of miR-135a, T-bet, and GATA-3 was quantified by real-time quantitative polymerase chain reaction (RT-qPCR). We utilized specific target gene primers purchased from Invitrogen Biotechnology Co. (China) for miR-135a (#MIMAT0000147), T-bet (#NM019507.2), and GATA-3 (#NM008091) in conjunction with FastStart Universal SYBR Green Master Mix (Rox) according to the manufacturer’s protocol (Roche Applied Science, Indianapolis, IN, USA). A SLAN® Real-Time PCR Detection System (Shanghai Hongshi Medical Technology Co., Ltd, China) was used with the following cycling conditions: denaturation at 95°C for 10 min, followed by 40 cycles of 95°C for 15s and 60°C for 60 s. A final extension was performed at 60°C for 5 min. Data were analyzed using the 2^−ΔΔCT^ method as previously described [14].

### Flow cytometric analysis

In Th cells, the transcription factors T-bet and GATA-3 are specific to Th1 and Th2, respectively [15–17]. Thus, we regarded CD4^+^T-bet^+^ and CD4^+^GATA-3^+^ T cells as Th1 and Th2 cells, respectively, in our analysis [18]. All reagents for this analysis were obtained from eBioscience (San Diego, CA, USA) unless otherwise noted. For detection of Th1 and Th2 cells by flow cytometry, a single-cell suspension was obtained from half of the spleen from each mouse and resuspended at 1 × 10^6^ cells/ml. This suspension was then incubated with 1 μl of anti-mouse CD4-FITC (0.5 mg/ml; #11–0041) for 30 min. Unbound antibody was then washed away with flow cytometry staining buffer twice at 1500 × rpm for 5 min. Samples were permeabilized with a fixation/permeabilization working buffer for 1 h and then washed with permeabilization buffer twice at 2500 × rpm for 5 min. Next, the samples were incubated with 1 μl of purified anti-mouse CD16/CD32 (0.5 mg/ml; #14–0161) as an Fc block for 15 min, and then stained with 3 μl of anti-mouse T-bet-PE (0.2 mg/ml; #12–5825) or 5 μl of anti-mouse GATA-3-PE antibody (0.06 μg; #12–9966) for 30 min. All incubations and washes were performed at 4°C. Cells were analyzed on a FACScan flow cytometer (BD Biosciences, San Jose, CA, USA).

### Histology

Mice were painlessly sacrificed with an intraperitoneal injection of 1% pentobarbital sodium 24 h after the last intranasal sensitization treatment. The heads of the mice were then removed and fixed with 4% paraformaldehyde overnight, followed by decalcification in an EDTA decalcifying solution for two weeks. The specimens were dehydrated in a series of increasing concentrations of ethanol and embedded in paraffin. Coronal sections (5 μm thick) of the nasal mucosa were obtained and histologically analyzed with hematoxylin and eosin (H&E) and MC staining to visualize eosinophils and MCs. These two cell types were counted in five randomly selected high-power fields (HPFs) at 400× magnification.

### Laser scanning confocal microscopy

Laser scanning confocal microscopy was used to visualize the localization of LV-mmu-miR-135a in MCs. Sections of the nasal mucosa obtained above were dried in a 60°C drying oven for 30 min, deparaffinized, and rehydrated using a standard protocol. The specimens were then incubated in an EDTA-Tris antigen retrieval solution at 90°C for 30 min. After recovery at room temperature, specimens were blocked with 10% normal goat serum for 1 h at room temperature and immunostained with a monoclonal rabbit anti-mouse MC tryptase antibody (1:100; #ab134932; Abcam, Cambridge, MA, USA) overnight at 4°C. IgG isotype controls were used for each group of specimens at the same concentration. The sections were then washed in phosphate-buffered saline (PBS) three times and labeled with fluorescently tagged goat anti-rabbit secondary antibody (1:100; #GB21303, Wuhan Google biotechnology Co., Ltd, Wuhan, China) for 1 h in darkness at room temperature. Sections were also incubated with the fluorescent nuclear dye DAPI (1:100; #C1002, Beyotime Biotech, Jiangsu, China) to determine the specific location of LV-mmu-miR-135a expression. The sections were again washed with PBS three times, and then mounted and sealed with antifade mounting medium (#G1401, Wuhan Google biotechnology Co.).

The nasal mucosa sections were evaluated within 24 h of staining using a laser scanning confocal microscope (Olympus FV1200, Japan). LV-mmu-miR-135a localization was observed in HPFs at 400 × magnification.

### Statistical analysis

All data are presented as the mean ± SEM. Differences between groups were compared using the non-parametric Kruskal-Wallis test, followed by the Mann-Whitney U test. Statistical calculations were performed using IBM SPSS Statistics 19.0 software (IBM, USA) and P < 0.05 was considered significant.

## Results

### Effect of LV miR-135a on serum IgE levels in AR mice

**Fig 2:**
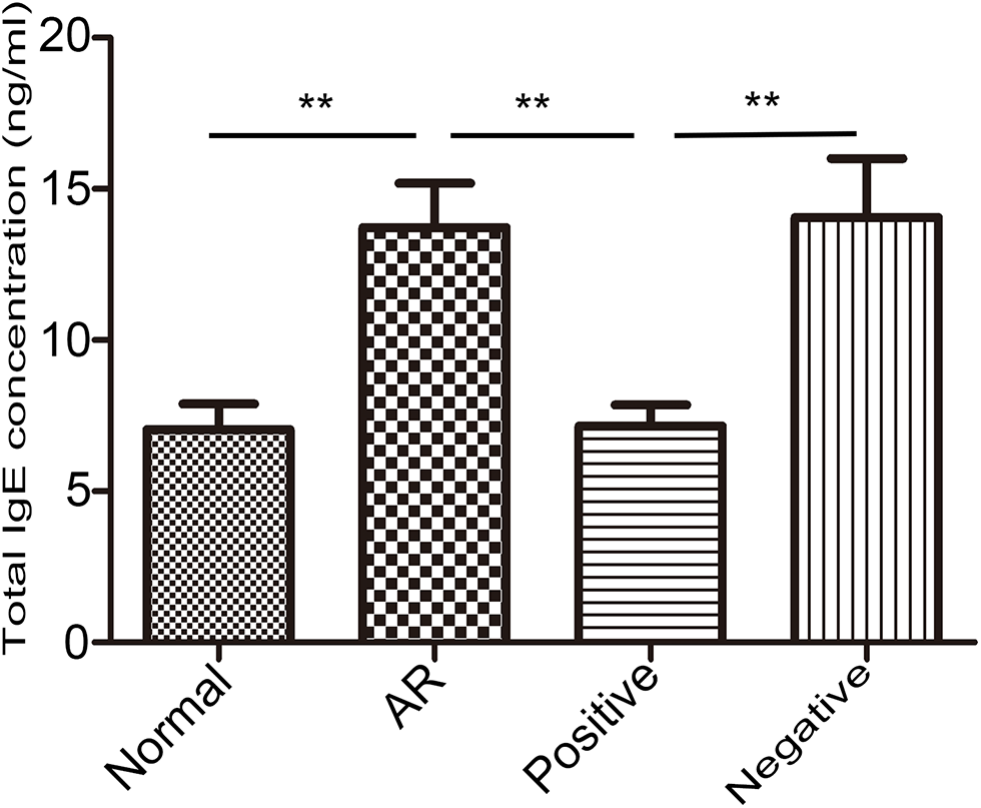
Effect of lentiviral-mmu-miR-135a on the immune response in AR mice. Total serum IgE concentrations were determined for the normal (control), AR (AR-induced), positive (AR-induced, treated with lentiviral-mmu-miR-135a), and negative (AR-induced, treated with empty lentivirus) groups 24 h after the final intranasal sensitization using ELISA. Data are presented as the mean ± SEM. **P<0.01.

To determine the effect of LV miR-135a on the allergic response observed in the AR mice, we measured the level of serum IgE (Fig 2). The level of total IgE in the serum was significantly upregulated in the AR mice compared to the normal mice. However, AR mice treated with LV miR-135a (the positive group) had levels of IgE similar to those of mice in the normal group, indicating that LV miR-135a blocks or reduces the immune response in AR mice. No significant difference in IgE levels was observed between the AR group and the negative group treated with an empty LV vector.

### Expression of miR-135a in the nasal mucosa of AR mice following LV infection

**Fig 3:**
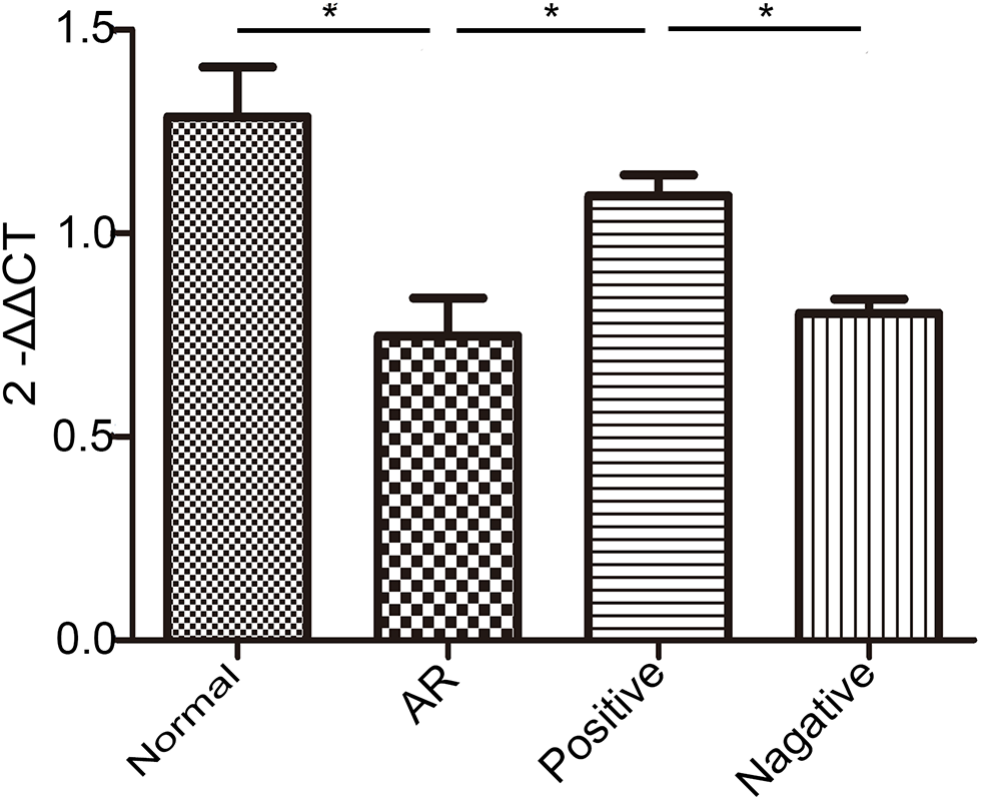
Expression of miR-135a in the nasal mucosa of AR mice following treatment with lentiviral-mmu-miR-135a. RT-qPCR was used to evaluate the relative expression levels of miR-135a in the nasal mucosa of mice in the normal (control), AR (AR-induced), positive (AR-induced, treated with lentiviral-mmu-miR-135a), and negative (AR-induced, treated with empty lentivirus) groups. Data are presented as the mean ± SEM. *P<0.05.

In our previous study, we found that miR-135a specifically base pairs to the 3′-UTR of GATA-3 mRNA and is downregulated during AR in our mouse model ^12^. To further understand the role of miR-135a during AR in the present investigation, we sought to overexpress miR-135a in the nasal mucosa of AR-induced mice via intranasal infection with an LV vector containing miR-135a. Notably, after AR mice were treated with LV miR-135a (positive group), miR-135a was significantly upregulated, indicating successful LV-host gene transduction in the nasal passages of these mice (Fig 3). In contrast, miR-135a expression in the negative and AR groups was significantly lower than that in the normal group and positive groups.

### Effect of LV miR-135a on cytokine expression in AR mice

**Fig 4:**
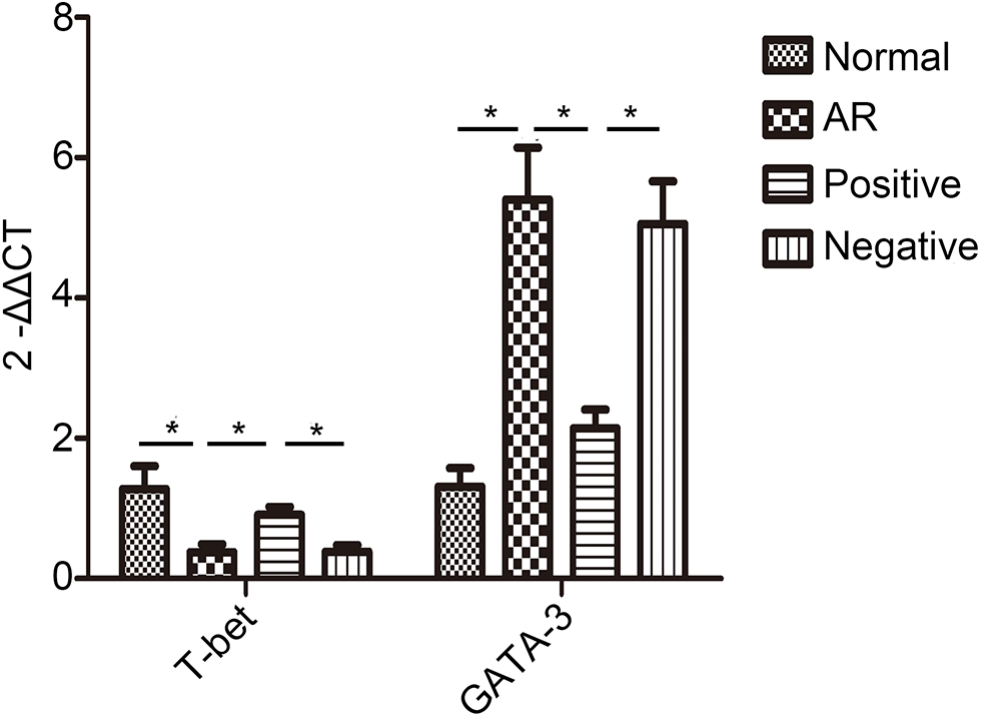
Overexpression of miR-135a alters T-bet and GATA-3 mRNA expression in AR mice. RT-qPCR was used to determine the relative mRNA expression of T-bet and GATA-3 in the nasal mucosa of normal (control), AR (AR-induced), positive (AR-induced, treated with lentiviral-mmu-miR-135a), and negative (AR-induced, treated with empty lentivirus) mice. Data are presented as the mean ± SEM. *P<0.05.

To better understand the effect of miR-135a overexpression on the nasal mucosa of AR mice, we evaluated the expression of T-bet and GATA-3 mRNA (Fig 4). The level of T-bet mRNA expressed in the nasal mucosa of the positive group was significantly higher than that of the AR and negative groups. Moreover, no difference was found between the positive and normal groups, whereas the level in the normal group was significantly higher than that in the AR and negative groups, indicating that treatment with LV miR-135a restored the expression of T-bet to a normal level in AR-induced mice. In contrast, GATA-3 mRNA expression was significantly decreased in the positive group compared to the elevated levels measured in the AR and negative groups, indicating suppression of the Th2-biased response. This miR-135a-mediated decrease in GATA-3 expression in the positive group also appeared to reestablish the normal physiological expression of this gene as there was no significant difference between the positive and normal groups.

### Flow cytometric analysis of Th cell polarization following LV miR-135a treatment

**Fig 5:**
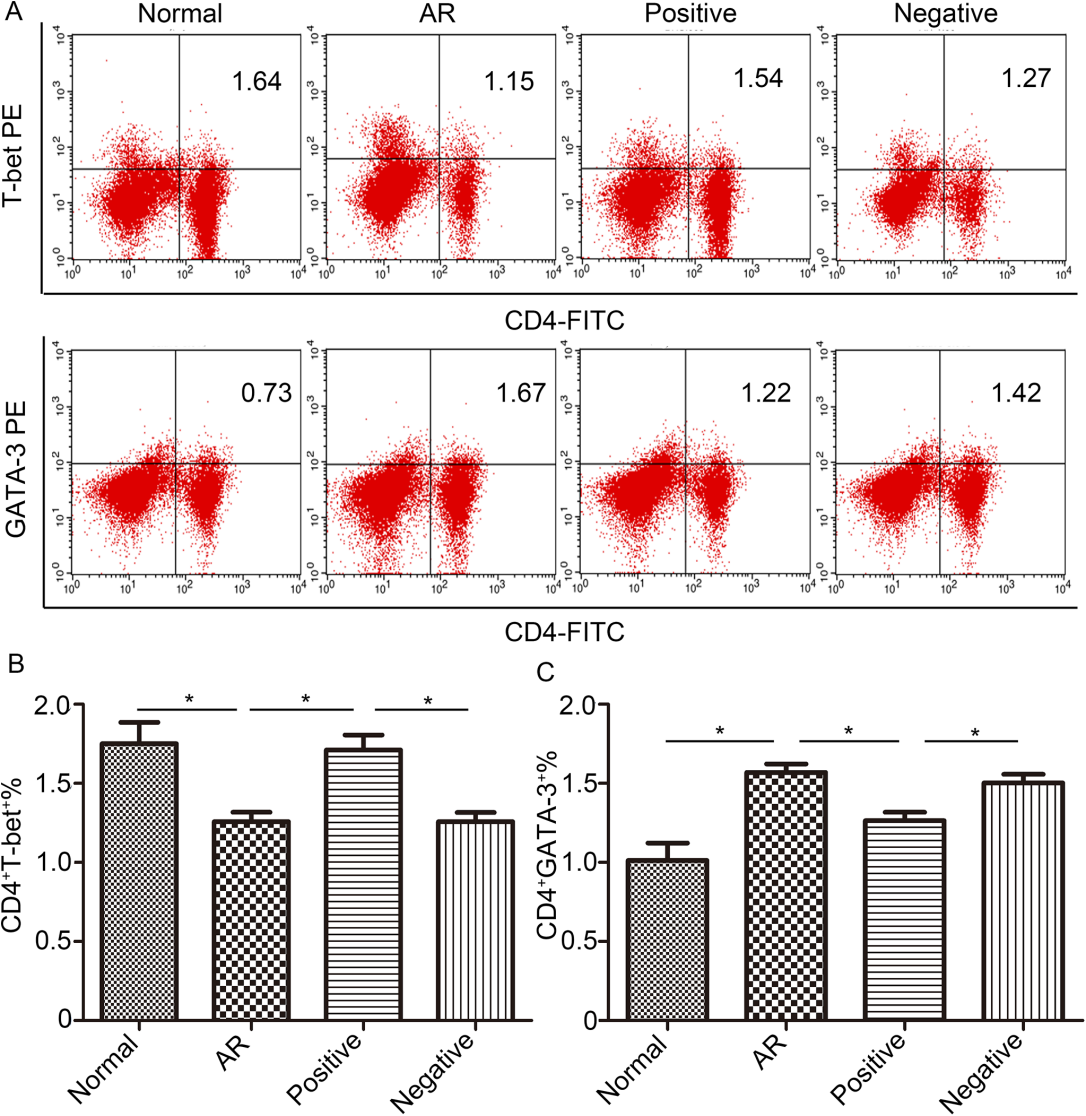
Lentiviral-mmu-miR-135a treatment influences Th cell polarization. The expression of T-bet and GATA-3 protein in CD4^+^ T cells was measured in the spleens of normal (control), AR (AR-induced), positive (AR-induced, treated with lentiviral-mmu-miR-135a), and negative (AR-induced, treated with empty lentivirus) mice using flow cytometry. (A) Representative dot plots from each experimental group. The percentages of CD4^+^T-bet^+^ T cells (B) and CD4^+^GATA-3^+^ T cells (C) were also calculated. Data are presented as the mean ± SEM. *P<0.05.

Th cell polarization, or the ratio of Th1 cells to Th2 cells, was measured by cytometrically counting the number of CD4^+^T-bet^+^ and CD4^+^GATA-3^+^ T cells in spleen samples from each mouse (Fig 5A). As shown in Fig 5B, the percentage of CD4^+^T-bet^+^ T cells in the negative and AR groups was significantly lower than that of the normal group but significantly higher in the positive group compared to the AR and negative groups, with levels being restored to normal. The percentage of CD4^+^GATA-3^+^ T cells in the negative and AR groups, in contrast, was significantly higher than that of the normal group (Fig 5C). Notably, treatment with LV miR-135a in the positive group appeared to significantly decrease this percentage back to a level similar to that of the normal group. Thus, the Th1/Th2 ratio (T-bet^+^ cells/GATA-3^+^ cells) for the AR group indicates a Th2 bias, as expected, and treatment with LV miR-135a appears to decrease this ratio back to normal physiological levels.

### Histological analysis of the nasal mucosa

**Fig 6:**
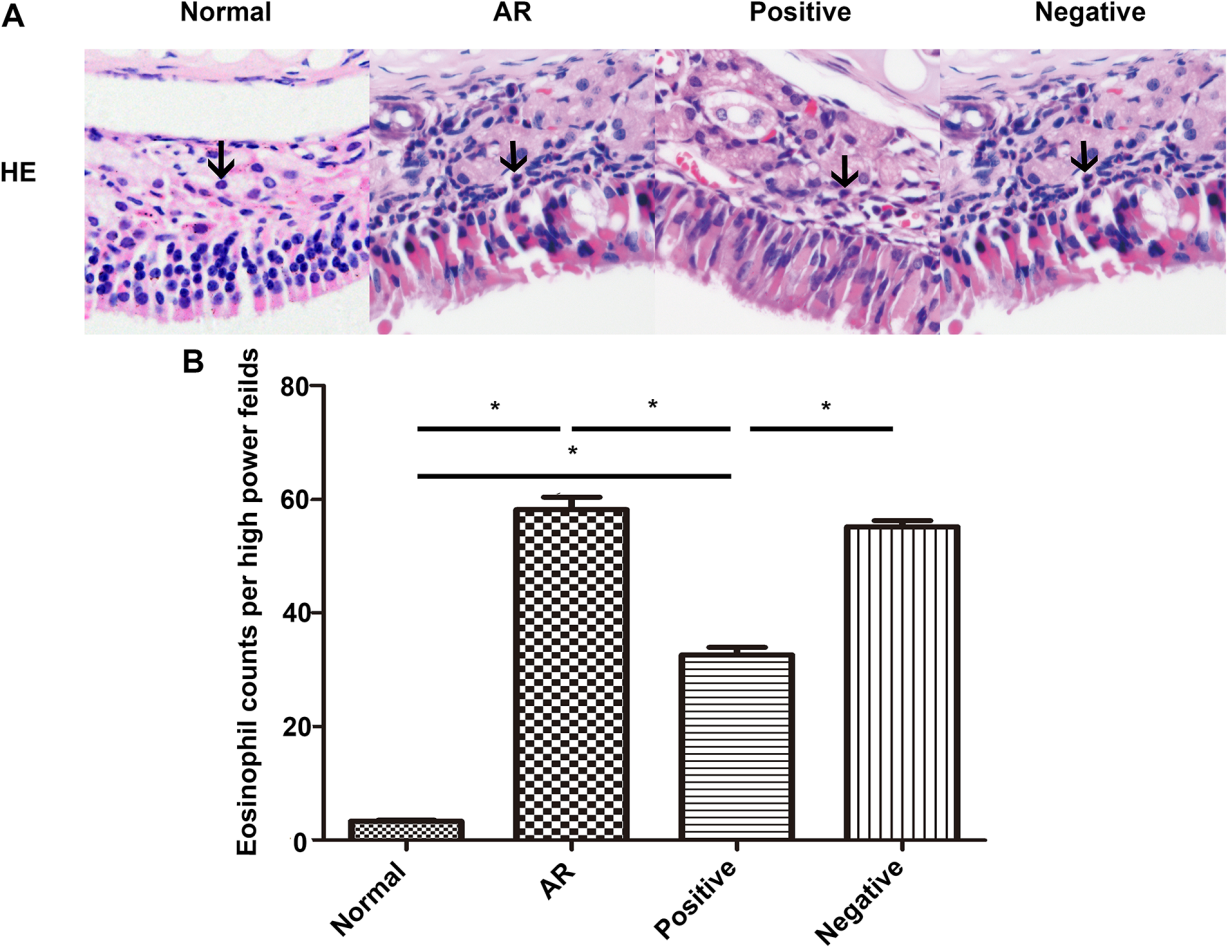
H&E staining of the nasal mucosa. The nasal mucosa of mice in the normal (control), AR (AR-induced), positive (AR-induced, treated with lentiviral-mmu-miR-135a), and negative (AR-induced, treated with empty lentivirus) groups was analyzed 24 h after the last OVA stimulation using H&E staining. (A) Representative images (original magnification × 400) of H&E stained eosinophils (black arrows) present in sections of the nasal mucosa from each experimental group. The number of eosinophils (B) was quantified for each group. Data are presented as the mean ± SEM. *P<0.05.

**Fig 7:**
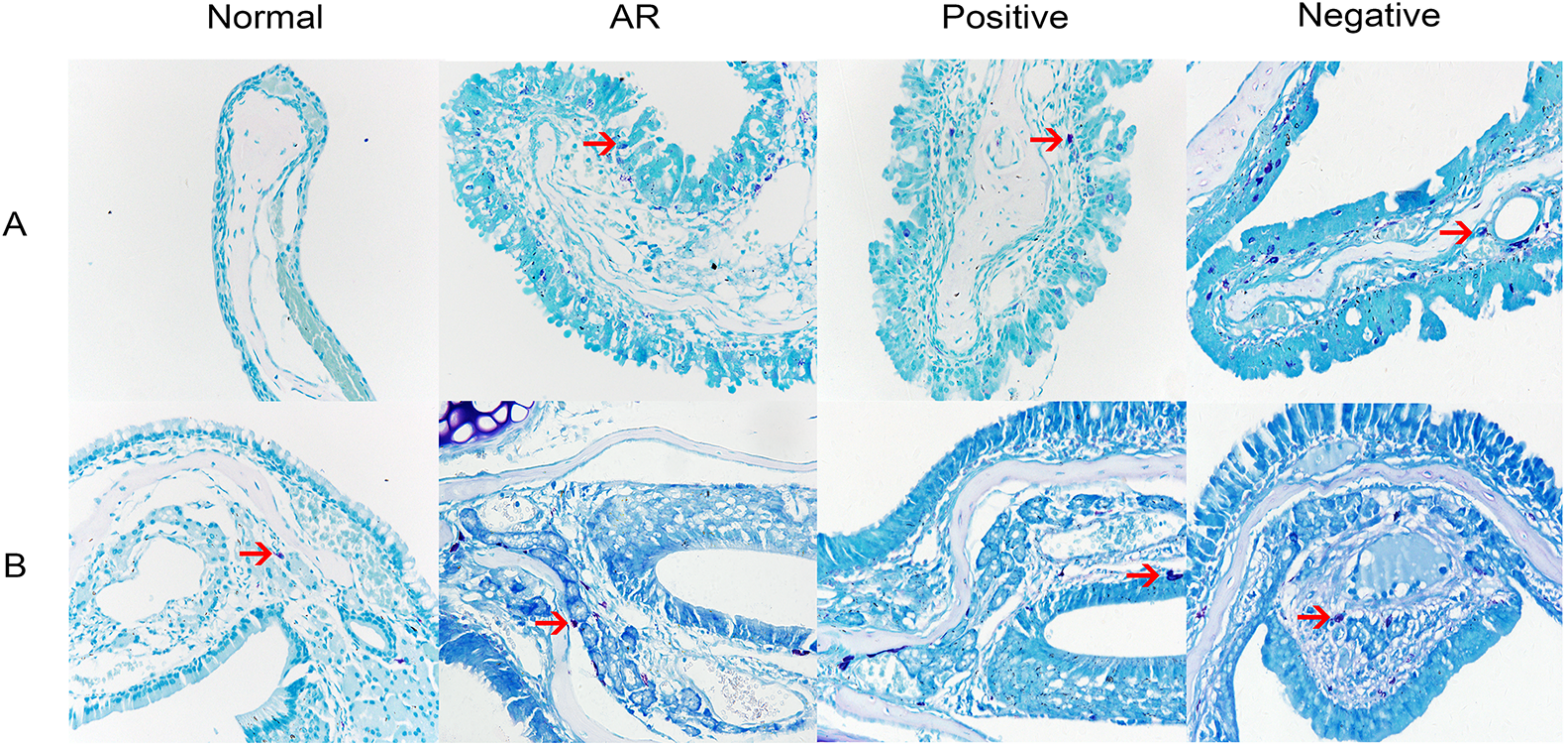
Mast cell staining of the nasal mucosa. The nasal mucosa of mice in the normal (control), AR (AR-induced), positive (AR-induced, treated with lentiviral-mmu-miR-135a), and negative (AR-induced, treated with empty lentivirus) groups was analyzed 24 h after the previous OVA stimulation using MC staining. Representative images (original magnification: 400×) of the stained MCs (red arrows) present in sections of the nasal mucosa from each experimental group. A is the respiratory region of the nasal mucosa, B is the olfactory region of the nasal mucosa.

Increased infiltration of leukocytes is a primary index used to diagnose AR. Thus, to evaluate the effect of miR-135a on the histological profile of our OVA-induced AR mouse model, we stained the nasal mucosa with H&E and MC staining solutions. The cytoplasm of the eosinophils was stained red (Fig 6A), whereas the cytoplasm of the MCs was stained purple (Fig 7). Notably, there was a significant influx of eosinophils (Fig 6B) and MCs (Table 1) in the nasal mucosa of mice in the AR group after the final intranasal treatment compared to that observed for the normal group, confirming nasal sensitization. Furthermore, although mice in the positive group had a significantly reduced number of eosinophils in their nasal mucosa compared to the AR and negative groups, they still had significantly more eosinophils than the normal group. In addition, in the epithelial layer, the number of MCs in the AR, the positive group, and the negative group was significantly higher than that in the normal group, but not significantly different across the three groups. The mice in the positive group showed a significantly reduced number of MCs in their lamina propria compared to the mice in the AR and negative groups, which still presented significantly more MCs than did the normal group. Additionally, in the respiratory region of the nasal mucosa, the number of MCs in the epithelial layer was significantly higher than that in the lamina propria (Fig 7A). However, in the olfactory region of the nasal mucosa, the number of MCs in the epithelial layer was significantly lower than that in the lamina propria (Fig 7B).

**Table 1:** MCs counts per high power field.

Group	A		B	
	Epithelial layer	Lamina propria	Epithelial layer	Lamina propria
**Normal**	0	0	0	1.00±1.41
**AR**	11.75±4.86	7.25±2.22	1.75±1.71	11.00±2.58
**Positive**	8.50±4.04	4.75±0.96	1.25±1.50	4.00±1.83
**Negative**	10.25±3.59	7.75±1.89	1.50±1.29	9.25±2.22

A is the respiratory region of the nasal mucosa, B is the olfactory region of the nasal mucosa. Data are presented as the mean ± SEM.

### MC localization of LV miR-135a

**Fig 8:**
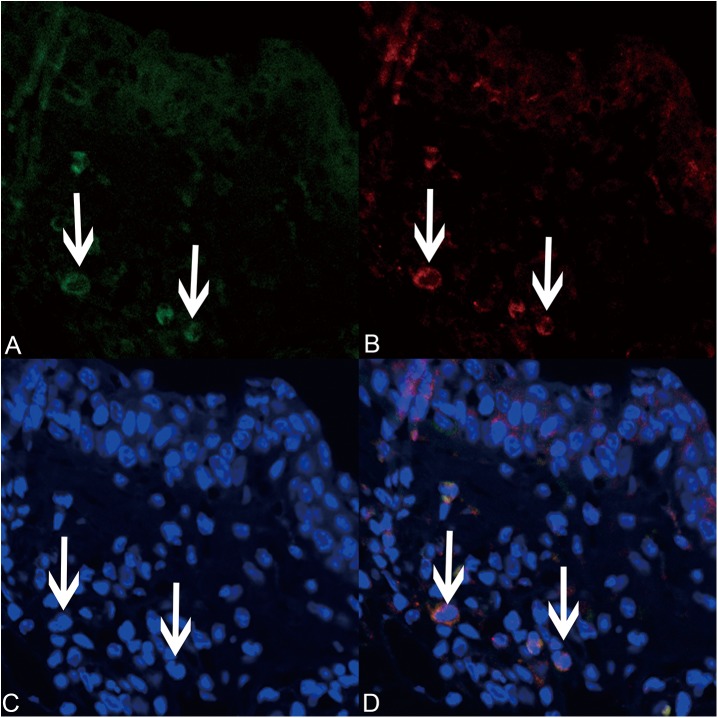
Lentiviral-mmu-miR-135a is expressed in mast cells. Laser scanning confocal microscopy (original magnification × 400) was used to analyze the expression of lentiviral-mmu-miR-135a (green; A) and tryptase (red; B) in mast cells present in the nasal mucosa of the positive group. Cell nuclei were stained with DAPI (blue; C). The merged image (D) highlights the overlapped green and red signals around the mast cell nuclei.

Laser scanning confocal microscopy was used to determine whether LV miR-135a localized specifically in the MCs of treated AR mice. As shown in Fig 8, the expression of miR-135a in the nasal mucosa after treatment with the LV miR-135a vector overlapped with expression of tryptase. These results suggest that LV miR-135a expression is MC.

## Discussion

In the present study, we investigated the effects of LV miR-135a on the allergic response that occurs during OVA-induced AR-related inflammation in a mouse model. Inflammation in any tissue is known to involve the activation of eosinophils and degranulation of MCs, which in turn release a variety of pro-inflammatory mediators that can cause tissue damage [2,19,20] and trigger the expression and secretion of various biologically active chemicals [3,21,22]. For example, we previously found that stimulation with OVA increases the concentration of interleukin 4 (IL-4) [11], reflecting the symptoms of an allergic reaction similar to that observed in AR patients. In the present study, we confirmed that OVA increases the infiltration of eosinophils and MCs in the nasal mucosa in addition to increasing the total serum IgE concentration and Th2 cell differentiation in our AR mouse model. Treatment with LV miR-135a strongly suppressed this allergic reaction.

Currently, the treatment options for patients with AR include allergen avoidance, pharmacotherapy, and immunotherapy. Recently, many researchers have indicated that miRNAs are relatively stable compared to many other mRNA moieties and even some proteins. This stability could be exploited to clinically modulate gene expression in a number of diseases, including AR and allergen-induced inflammation [23,24]. In fact, several studies have investigated the therapeutic potential of various miRNAs specifically during the Th1/Th2 immune response [25–28]. For example, miR-21 upregulates IL-12 in the airway during an allergic reaction by binding to IL-12p35 and increasing interferon (IFN)-γ expression, which in turn downregulates IL-4 and promotes a Th1 bias [26]. Thus, targeting of miR-21 by gene therapy could help to treat this specific type of allergic response. Furthermore, miR-155-deficient Th cells also have a Th2 bias *in vitro* that appears to be caused by changes in the targeted transcription of v-Maf musculoaponeurotic fibrosarcoma oncogene homolog (c-Maf), resulting in the upregulation of IL-4 [27,28]. MiR-126 has also been found to be upregulated in the airway of an asthma mouse model, and its suppression reduces inflammation, expression of Th2 cytokines (e.g., IL-5 and IL-13), and airway hyperreactivity [29]. Although each of these specific miRNAs, in addition to many others, could potentially be used as drug targets in allergen-induced inflammation, current research regarding the actual therapeutic effects of these miRNAs during AR *in vivo* is limited.

Furthermore, although miRNA toxicity is likely minimal [30], the delivery vehicles utilized often cause detrimental side effects, such as unexpected cellular differentiation and inappropriate activation of the immune response [31]. Thus, to avoid these complications, we utilized LV transduction vectors to intranasally infect OVA-sensitized AR mice with our miRNA of interest. LV vectors have been used to infect various types of cells while carrying exogenous genes, successfully integrating the transgenes into the genome of the host cell for long-term stable expression [32]. Moreover, these LV vectors do not transfer their viral proteins, making them the ideal miRNA delivery vehicle [33]. To overcome possible systemic symptoms that could be caused by tail vein injection of LV vectors, and to enhance the LV transfection efficiency in the nasal mucosa, we intranasally administered our miRNA-containing LV vector to specifically infect the nasal mucosa (through nasal drip) and investigate the directed therapeutic effects on this tissue during AR. This type of directed treatment using an LV vector has been implemented previously in the trachea, where intratracheally administering an LV vector containing GATA-3 small hairpin RNA (shRNA) resulted in successful transduction of cells, specifically in the lungs [13].

Our decision to focus on the therapeutic potential of miR-135a stems from our previous work indicating that this miR-135a can specifically bind to GATA-3 and that overexpression of miR-135a in AR mice decreases the mRNA and protein expression of both GATA-3 and IL-4 [11]. Here, these data were confirmed and we further highlighted the ability of this miR-135a to decrease GATA-3 mRNA and protein expression and increase T-bet mRNA expression in the nasal mucosa. In addition, enforced GATA-3 expression was sufficient to enhance the activity of Th2 and group 2 innate lymphoid cells (ILC2), leading to increased susceptibility to allergic airway inflammation [34,35]. In our study, treatment the mice with LV miR-135a restored the expression of T-bet to a normal level in AR-induced mice. In contrast, GATA-3 mRNA expression was significantly decreased in the positive group compared to the AR and negative groups, indicating suppression of the biased Th2 response. Therefore, LV miR-135a could reduce the infiltration of eosinophils and MCs in the nasal mucosa and reestablish balanced Th1/Th2 differentiation, possibly through a direct effect on ILC2s and Th2 cells.

In our study, we found that LV miR-135a significantly decreased the number of MCs in the lamina propria tissue in the positive group, but the number of MCs in the epithelial layer was not significantly different across the AR and positive and negative groups. Amin [2] indicated that the number of MCs increases at sites of inflammation. To reach these areas, MCs or their progenitors could migrate, or resident MC precursors could be activated and proliferate. We hypothesized that miR-135a deceased the activity of MCs; however, because the inflammatory response in the epithelial layer could still be detected, the MCs in the lamina propria were still active and migrated into the epithelial layer. Thus, the number of MCs in the lamina propria was significantly decreased by miR-135a. In allergic inflammation, the epithelial layer of the respiratory region of the nasal mucosa is the initial injury site; therefore, there were more MCs here than in the lamina propria. MCs are localized in connective tissue and the lamina propria of the olfactory region of the nasal mucosa is near connective tissue; therefore, it contained more MCs than the epithelial layer. Further studies are required to test these hypotheses.

Laser scanning confocal microscopy indicated that our vector not only successfully transfected the nasal mucosa but also specifically infected the MCs, as shown by the overlap of miR-135a expression with expression of tryptase, a known marker of mature MCs [36,37]. Taking into consideration the relationship between miR-135a and GATA-3 [11] and the role of GATA-3 in MC regulation and differentiation [3,4], we predict that miR-135a reduces MC infiltration and degranulation during AR via targeted GATA-3 binding. However, the specific signaling mechanism remains unclear, and requires further exploration. The toxic effects of LV miR-135a were also minimal and H&E staining of lung tissue did not show any significant histomorphological changes (S1 Fig). In fully awake mice, when reflexes are active, fluid applied to the nares is not easily inhaled into the lower respiratory tract. Inflammatory conditions of the upper airways are well known to be associated with lower airway disease [38].The resolution of allergy inflammation via LV miR-135a treatment may be responsible for the beneficial effect of the treatment on lower airway disease. Additional clinical trials utilizing LV miR-135a should be performed to determine the full therapeutic potential of this specific miRNA.

## Conclusion

Overall, the present study has demonstrated that LV miR-135a can be used to correct Th1/Th2 imbalances in the nasal mucosa and spleen of AR-induced mice. Administration of LV miR-135a also appears to strongly suppress the allergen-induced inflammation commonly observed in AR mice, which includes increased total serum IgE concentrations, infiltration of eosinophils and MCs in the nasal mucosa, and increased GATA-3 expression, all of which returned to normal levels following treatment. It is likely that LV miR-135a-mediated changes in MCs via direct targeting of GATA-3. Although additional work is necessary to fully elucidate the function of miR-135a during AR, this study provides a foundation for the use of intranasally administered miRNA-containing LV vectors for treatment of allergen-induced inflammation.

## Supporting Information

S1 Fig H&E staining of the lung. A is a representative image (magnification:100×) of a lung in the positive group; B is a representative image (magnification:100×) of a lung in the negative group.
(TIF)
