# Supplementary figures and images for: Intranasal Administration of Lentiviral miR-135a Regulates Mast Cell and Allergen-Induced Inflammation by Targeting GATA-3
Source: PLoS One. 2015 Sep 29;10(9):e0139322. doi: 10.1371/journal.pone.0139322 (PMC4587974; doi:10.1371/journal.pone.0139322)

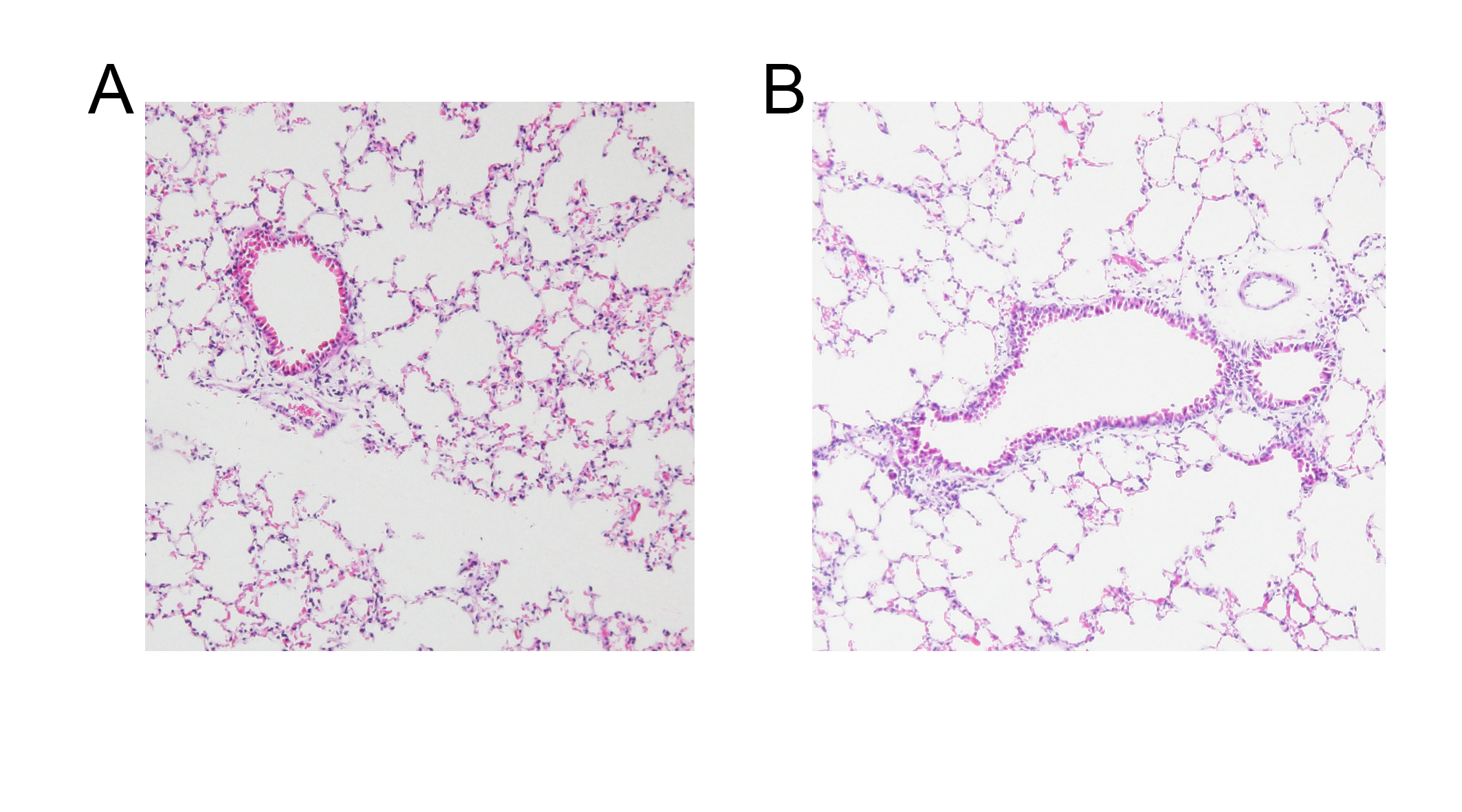

Supplement: S1 Fig — A is a representative image (magnification:100×) of a lung in the positive group; B is a representative image (magnification:100×) of a lung in the negative group. (TIF) [file pone.0139322.s001.tif]
